# Peroneus Longus Tendon Versus Hamstring Tendon Autograft for Primary Anterior Cruciate Ligament (ACL) Reconstruction: A Systematic Review and Meta-Analysis

**DOI:** 10.7759/cureus.105983

**Published:** 2026-03-27

**Authors:** Shahmeen Rasul, Osasenaga Bencharles, Maira Muqqaddam, Ranj Bhakar, Shashwat Shetty, Arnov Mukherjee, Siddhesh V Kulkarni, Farhan Saleem

**Affiliations:** 1 Trauma and Orthopaedics, University Hospital of Derby and Burton NHS Foundation Trust, Derby, GBR; 2 Medicine and Surgery, University Hospital of Derby and Burton NHS Foundation Trust, Derby, GBR; 3 Internal Medicine, NHS University Hospitals of Liverpool Group, Liverpool, GBR; 4 Trauma and Orthopaedics, Torbay Hospital, Torquay, GBR; 5 Orthopaedics, Hillingdon Hospital, Uxbridge, GBR; 6 Trauma and Orthopaedics, Queens Hospital Burton, Burton, GBR; 7 Trauma and Orthopaedics, Hillingdon Hospitals NHS Foundation Trust, London, GBR; 8 Orthopaedic Surgery, Lahore General Hospital, Lahore, PAK

**Keywords:** anterior cruciate ligament reconstruction, graft selection, hamstring tendon autograft, meta-analysis, peroneus longus tendon autograft

## Abstract

Anterior cruciate ligament (ACL) reconstruction remains one of the most commonly performed orthopedic procedures worldwide, with graft selection representing a key determinant of postoperative outcomes. In recent years, the peroneus longus tendon has emerged as a promising alternative to the traditionally used hamstring tendon autograft. Previous meta-analyses have suggested comparable or favorable outcomes; however, several new randomized controlled trials and observational studies have since been published, necessitating an updated synthesis of the evidence.

This systematic review and meta-analysis, therefore, aims to comprehensively evaluate and compare the clinical, functional, and stability outcomes of peroneus longus tendon versus hamstring tendon autografts in primary ACL reconstruction.

A systematic literature search was conducted across PubMed, Cochrane Library, Web of Science, and Embase from inception until January 2026. Studies comparing the peroneus longus tendon with hamstring tendon autografts reporting at least one predefined outcome were eligible for inclusion. A total of 32 studies comprising 10 randomized controlled trials and 22 observational studies were included. Pooled analyses were performed using random-effects models, with outcomes reported as mean differences (MDs) or odds ratios with 95% confidence intervals.

No statistically significant differences were observed between the two groups in terms of International Knee Documentation Committee (IKDC) score (MD: -0.74, 95% CI: -1.58 to 0.09) or Lysholm score (MD: -0.50, 95% CI: -1.17 to 0.18). Additionally, objective knee stability measures, including the anterior drawer, Lachman, and pivot shift tests, were also not significantly different between the two groups. Subgroup analysis of randomized controlled trials demonstrated significantly higher IKDC and Lysholm scores in the peroneus longus tendon group. Patients in the hamstring tendon group exhibited significantly greater thigh circumference reduction compared to the peroneus longus tendon group (MD: 0.92 cm, 95% CI: 0.72 to 1.11), indicating more pronounced donor site muscle atrophy. Ankle function, assessed by American Orthopaedic Foot and Ankle Society (AOFAS) scores, remained comparable between groups.

Peroneus longus tendon autograft demonstrates comparable functional and stability outcomes to hamstring tendon autograft while offering superior preservation of thigh muscle mass. These findings support its consideration as a viable alternative graft option in primary ACL reconstruction, particularly in patients where minimizing knee donor site morbidity is a priority.

## Introduction and background

Injuries to the anterior cruciate ligament (ACL) are among the most frequent and functionally limiting knee injuries, occurring at an estimated rate of 68.6 cases per 100,000 person-years in the general population [1]. The burden of ACL injury extends beyond the immediate trauma: those who sustain an ACL injury face up to a 10-fold higher risk of developing early-onset degenerative knee osteoarthritis, with approximately 50% of affected individuals reporting knee osteoarthritis with associated pain and functional impairment at 10 to 20 years following injury [2]. Beyond long-term joint degeneration, reinjury rates remain a critical concern. Among athletes under 20 years of age who return to high-risk sports, approximately one in five sustains a reinjury to either knee following ACL reconstruction (ACLR). These outcomes carry substantial personal, athletic, and socioeconomic consequences, underscoring the clinical imperative to optimize both operative technique and graft selection [2].

For physically active patients aiming to regain their pre-injury level of performance, ACLR is widely regarded as the preferred treatment approach, with more than 400,000 procedures carried out globally each year [2]. The overall outcome of ACLR is strongly influenced by factors such as graft selection, operative technique, and postoperative rehabilitation, and the optimal choice of graft continues to be debated within the orthopedic community. Graft selection for ACLR is largely based on the surgeon's experience and preference, although several patient-specific factors must be considered, including patient size, as hamstring tendons (HTs) may be too small to allow for an adequate graft, particularly in petite female patients [3].

HT autografts - most commonly using the semitendinosus and gracilis tendons - have been routinely employed in ACLR since the 1980s and remain one of the most commonly selected graft options worldwide [4]. Their reported benefits include reduced donor site morbidity, less anterior knee discomfort, smaller surgical incisions, and preservation of the extensor apparatus [5]. Pooled data from randomized controlled trials (RCTs) have shown no statistically significant differences between patellar tendon and hamstring grafts for functional assessment, return to activity, Tegner and Lysholm scores, or re-rupture rates, though all stability tests consistently demonstrated that patellar tendon reconstruction resulted in a more statically stable knee. Nonetheless, potential drawbacks of HT grafts - including postoperative hamstring weakness, increased graft laxity, and tunnel enlargement - have led clinicians to explore alternative autograft sources [6]. 

More recently, the peroneus longus tendon (PLT) has gained attention as a potential graft option for primary ACLR, first introduced by Rhatomy and colleagues in 2017 [7]. Anatomically situated in the lateral compartment of the lower leg, the PLT contributes to foot eversion and plantarflexion. Biomechanical evaluations indicate that it offers suitable properties for ACLR, including sufficient length (approximately 25-28 cm), adequate diameter (around 5-7 mm), and tensile strength comparable to or greater than that of the native ACL [8]. The ultimate strength of a doubled PLT graft has been shown to be significantly higher than that of the native ACL and comparable to a quadrupled hamstring construct, with dimensions that are more consistent than those of the HT. This dimensional consistency is a particularly meaningful advantage in surgical planning, as it reduces the risk of undersized graft constructs - a known contributor to graft failure, especially in younger and smaller patients. Additionally, harvesting the PLT may theoretically maintain knee proprioception and muscular strength while preserving both flexor and extensor mechanisms [9-10]. Unlike HT harvest, which frequently disrupts the hamstring-quadriceps balance and may compromise dynamic knee stability during rehabilitation, PLT harvest occurs entirely distal to the knee joint, with the peroneus brevis compensating for the loss of eversion and plantarflexion function [9].

Despite these proposed advantages, comparative clinical data between PLT and HT autografts remain sparse and variable. Some investigations have demonstrated encouraging short-term results with PLT grafts, showing acceptable knee stability, favorable functional outcomes, and low donor site morbidity [11]. Recent comparative data suggest that PLT autografts offer advantages, including a larger graft diameter, better preservation of knee flexion function, fewer symptoms related to the saphenous nerve, and excellent ankle outcomes relative to HT autografts. However, concerns have been expressed regarding possible ankle instability, risk of peroneal nerve injury, and the technical learning curve associated with PLT harvesting [12]. In contrast, HT autografts benefit from extensive long-term evidence and are often considered the reference standard when assessing newer graft alternatives [13].

In light of the growing adoption of PLT autografts and inconsistent findings across existing studies, a thorough systematic review and meta-analysis are needed to consolidate the available evidence. The present analysis seeks to compare the effectiveness and safety of PLT and HT autografts in primary ACLR, focusing on outcomes such as knee stability, functional performance scores, graft failure incidence, donor site morbidity, and overall complications. By integrating results from multiple studies, this meta-analysis aims to generate more robust evidence to support informed clinical decision-making and to clarify the advantages and disadvantages associated with each graft option in primary ACLR.

## Review

Methodology

This study was conducted according to the Preferred Reporting Items for Systematic Reviews and Meta-Analyses (PRISMA) guidelines [14].

Literature Search

A comprehensive and systematic search of four major electronic databases - PubMed, Cochrane Library, Web of Science, and Embase - was independently performed by two reviewers. The search spanned from database inception through 30 January 2026, capturing the full breadth of available evidence on this topic without imposing an arbitrary start date, thereby reducing the risk of missing historically important comparative studies. The rationale for selecting these four databases was to maximize coverage across clinical, biomedical, and evidence synthesis literature, as each database indexes distinct but partially overlapping bodies of research.

A structured Boolean search strategy was applied consistently across all databases, combining Medical Subject Headings (MeSH) terms with free-text keywords to ensure both sensitivity and specificity. The search string was constructed as follows: ("anterior cruciate ligament" OR "ACL") AND ("reconstruction") AND ("peroneus longus" OR "fibularis longus") AND ("hamstring" OR "semitendinosus" OR "gracilis").

Boolean operators were used deliberately: the OR operator was applied within each conceptual group to capture synonymous or related terms (e.g., "peroneus longus" and "fibularis longus" refer to the same anatomical structure under different nomenclature systems), while the AND operator linked the four conceptual domains - ligament, procedure, experimental graft, and comparator graft - to ensure retrieved records were relevant to all components of the research question. Two additional filters were applied uniformly across all databases: a species filter restricting results to human studies only, excluding animal and in vitro research, and a language filter limiting inclusion to English-language publications. To further minimize the risk of missing eligible studies, the reference lists of all included articles and relevant systematic reviews were manually screened for additional records not captured through electronic searching.

Study Selection

Eligible studies were required to meet the following criteria: (1) RCTs or observational designs (cohort or case-control studies) directly comparing PLT autografts with HT autografts in primary ACLR; and (2) reporting at least one of the prespecified outcome variables. Studies were excluded if they: (1) were non-clinical investigations (e.g., in vitro or animal studies); (2) evaluated allografts or hybrid/combined graft techniques; (3) did not have accessible full-text articles; (4) were published in languages other than English; or (5) involved multiligament knee injuries or revision ACL procedures.

Two reviewers independently screened the titles and abstracts of all retrieved records to determine potential eligibility. Full-text versions of studies deemed potentially relevant were subsequently reviewed in detail according to the predefined inclusion and exclusion criteria. Any discrepancies between the reviewers were settled through discussion, and when necessary, by consulting a third senior reviewer to reach consensus.

Data Extraction

Data were independently collected by two reviewers using a standardized extraction sheet developed specifically for this meta-analysis. From each eligible study, the following details were recorded: (1) study-related information, including first author, publication year, study design (RCT or observational study), and country or region of origin; (2) participant characteristics, such as total sample size, number of participants in each treatment group, mean age, and proportion of male patients; (3) duration of follow-up; and (4) reported outcome variables. Any inconsistencies in the extracted data were addressed through discussion between the two reviewers, with unresolved issues adjudicated by a third reviewer to achieve agreement.

Quality Assessment

The methodological rigor of the included studies was independently evaluated by two reviewers using design-specific appraisal tools.

For RCTs, the Cochrane Collaboration revised Risk of Bias tool (RoB 2) was applied. This instrument assesses potential bias across five domains: (1) bias arising from the randomization process; (2) bias due to deviations from intended interventions; (3) bias related to incomplete outcome data; (4) bias in outcome measurement; and (5) bias in the selection of reported results. Each domain was categorized as “low risk,” “some concerns,” or “high risk” of bias, leading to an overall risk-of-bias judgment for each trial [15].

For observational studies, quality was appraised using the Newcastle-Ottawa Scale (NOS). This scale examines three key domains: (1) selection of study groups (maximum 4 points); (2) comparability between groups (maximum 2 points); and (3) assessment of outcomes (maximum 3 points), yielding a total possible score of 9 points. Studies scoring 7-9 points were classified as high quality, those with 4-6 points as moderate quality, and those with 0-3 points as low quality. Any discrepancies in quality ratings were resolved through discussion between the two reviewers, and if necessary, by consulting a third senior reviewer [16].

Outcomes

Functional outcomes were evaluated using two validated knee-specific instruments. The International Knee Documentation Committee (IKDC) score assesses symptoms, functional status, and the ability to return to sports activities, with scores ranging from 0 (poorest function) to 100 (optimal function). The Lysholm Knee Scoring Scale is a patient-reported tool that measures knee-related symptoms and limitations in daily activities, also scored on a scale from 0 to 100, where higher values represent superior knee performance.

Donor site morbidity was assessed using the American Orthopaedic Foot and Ankle Society (AOFAS) ankle-hindfoot score. This instrument evaluates pain, functional capacity, and alignment, with total scores ranging from 0 to 100; higher scores indicate better ankle function.

Physical examination findings included thigh circumference, measured in centimeters at a standardized distance proximal to the patella to detect quadriceps or hamstring muscle atrophy after graft harvesting. Knee stability was assessed using the Lachman test, which evaluates anterior tibial translation and is graded as negative, grade 1+ (0-5 mm), grade 2+ (5-10 mm), or grade 3+ (>10 mm). The anterior drawer test, performed with the knee flexed at 90 degrees, was used to assess anterior laxity and graded in the same manner as the Lachman test. Rotational stability was examined using the pivot shift test, a dynamic maneuver graded as negative, grade 1+ (glide), grade 2+ (clunk), or grade 3+ (marked instability).

Data Analysis

All data analyses were conducted using the RevMan Version 5.4.1 (The Cochrane Collaboration, Oxford, UK). For outcomes including Lysholm Knee Scoring Scale, IKDC, thigh circumference, and AOFAS score, the mean difference (MD) was reported along with 95% confidence intervals (CI) using random-effect models. For outcomes including the Lachman test, anterior drawer test, and pivot shift test, the odds ratio (OR) was reported along with 95% CI using a random-effect model. An effect size with a P-value less than 0.05 was considered statistically significant. Heterogeneity between studies was assessed using the I² statistic. Subgroup analysis was performed for two outcomes, including IKDC and Lysholm Knee Scoring Scale, by reporting estimates at different timepoints, including six months, 12 months, and 24 months. Subgroup analysis for these outcomes was also performed based on study designs.

Results

**Figure 1:**
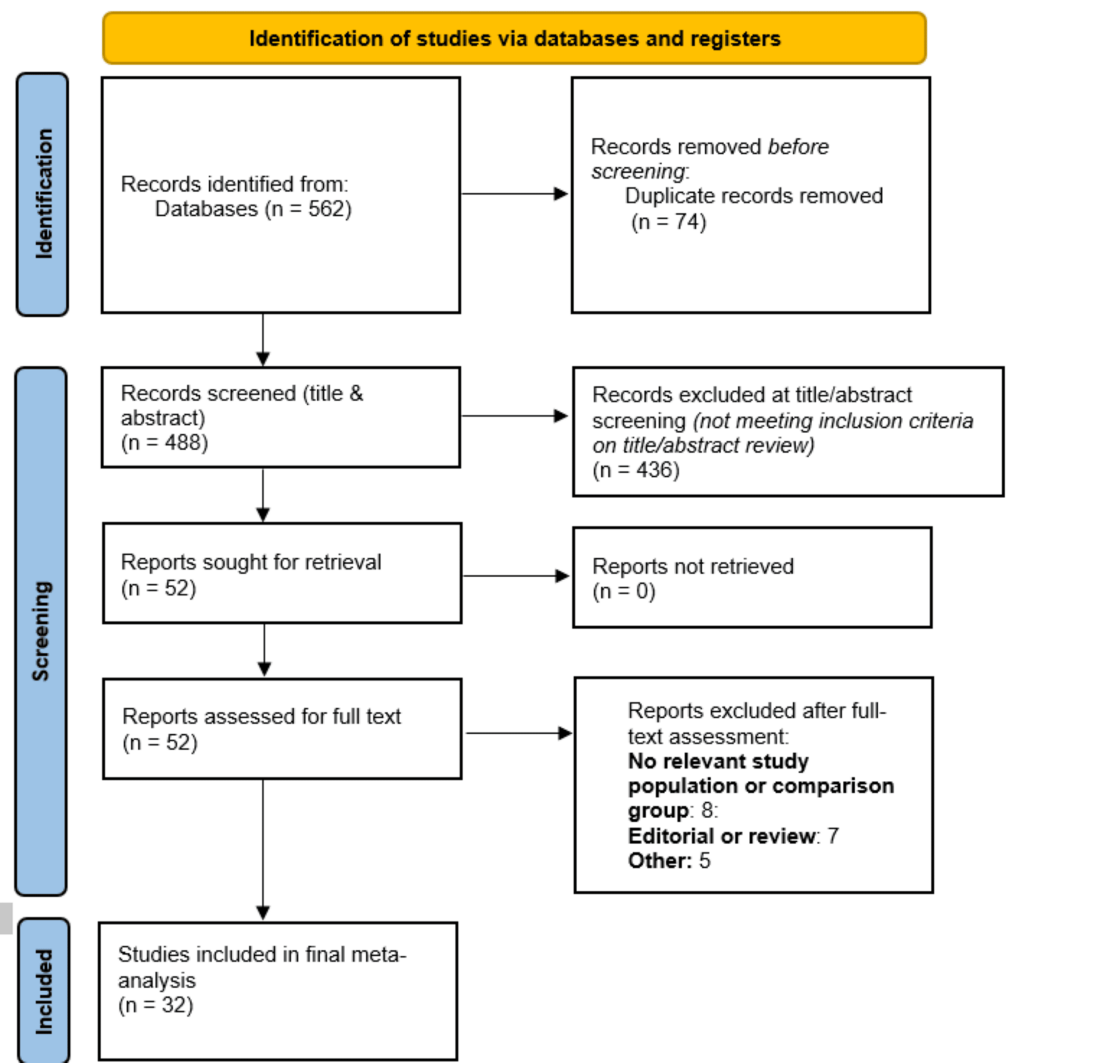
Preferred Reporting Items for Systematic Reviews and Meta-Analyses (PRISMA) flowchart (study selection process)

Figure 1 presents PRISMA showing the study selection process. Initial database screening yielded 562 studies. After removing duplicates, 488 studies were initially screened. Full-text screening of 52 studies was done. Finally, 32 studies were included in the meta-analysis. Table 1 presents the characteristics of included studies. Out of 32 included studies, 10 were RCTs while 22 were observational. The sample size of included studies ranged from 24 to 194. The majority of the studies were conducted in India (n = 16). Quality assessment of included observational studies and RCTs is shown in Table 2 and Table 3, respectively. All 10 RCTs demonstrated low risk in the randomization process. However, some concerns were noted in deviations from intended interventions and selective reporting in several studies. High risk of bias in outcome measurement was identified in four trials due to potential lack of blinding or subjective outcome assessment. Observational studies were evaluated using the NOS. Among the 22 included studies, 16 were rated as good quality and six as fair quality, with none classified as poor. Overall, the included studies demonstrated moderate to high methodological quality.

**Table 1:** Study characteristics of the 32 included studies This table summarizes the baseline characteristics of all 32 studies identified and included through the systematic database search conducted across PubMed, Cochrane Library, Web of Science, and Embase (inception to 30 January 2026), following PRISMA 2020 guidelines. Data were synthesized from randomized controlled trials and observational studies (prospective and retrospective cohort designs) that directly compared PLT autografts with HT autografts in primary anterior cruciate ligament reconstruction. Characteristics presented include first author, publication year, country of origin, study design, sample size per group, mean patient age, proportion of male participants, and mean follow-up duration. HT, hamstring tendon; NR, not reported; PLT, peroneus longus tendon; PRISMA, Preferred Reporting Items for Systematic Reviews and Meta-Analyses; RCT, randomized controlled trial References: [8,17-47]

Author	Year	Study Design	Region	Groups	Sample Size	Follow-Up	Mean Age	Males (n)
Acharya et al. [17]	2024	Observational	India	PLT	30	12 Months	27.73	28
				HT	30		28.56	26
Agarwal et al. [18]	2023	Observational	India	PLT	27	24 Months	NR	NR
				HT	27			
Agarwal et al. [19]	2023	Observational	India	PLT	98	12 Months	28	68
				HT	96		27.5	57
Arva et al. [20]	2026	Observational	India	PLT	38	6 Months	27.85	34
				HT	40		29.47	31
Ashtekar et al. [21]	2025	Observational	India	PLT	12	6 Months	29.92	NR
				HT	12		30.17	
Asif et al. [22]	2024	RCT	India	PLT	60	12 Months	24.4	NR
				HT	60		24.73	
Bi et al. [23]	2018	RCT	China	PLT	62	24 Months	29.1	34
				HT	62		27.9	31
Butt et al. [24]	2025	RCT	Pakistan	PLT	30	60 Months	27.73	30
				HT	30		29.17	29
Choudhari et al. [25]	2023	Observational	India	PLT	20	6 Months	30.9	17
				HT	20		33.1	15
Dwidmuthe et al. [26]	2024	RCT	India	PLT	18	6 Months	NR	NR
				HT	18			
Gök et al. [27]	2024	Observational	Turkey	PLT	52	20.6 Months	28	47
				HT	54		28.9	46
Gunadham et al. [28]	2022	Observational	Thailand	PLT	13	36 Months	27.6	10
				HT	39		31.1	38
Hussain et al. [29]	2025	RCT	Pakistan	PLT	39	3 Months	31.8	29
				HT	39		30.9	27
Kapoor et al. [30]	2025	Observational	India	PLT	28	9 Months	NR	25
				HT	28			26
Keyhani et al. [31]	2022	Observational	Iran	PLT	65	24 Months	29.8	58
				HT	65		27.6	61
Khalid et al. [32]	2024	Observational	Pakistan	PLT	40	24 Months	29	32
				HT	40		28	30
Khalil et al. [33]	2025	RCT	Egypt	PLT	36	18 Months	27.89	33
				HT	35		27.57	32
Ligu et al. [34]	2024	Observational	India	PLT	23	12 Months	33.57	17
				HT	22		31.82	18
Nikumbh et al. [35]	2023	Observational	India	PLT	40	32.1 Months	28.5	35
				HT	40		26.9	38
Punnoose et al. [36]	2024	Observational	India	PLT	30	12 Months	28.07	25
				HT	30		29.53	27
Rahul et al. [37]	2025	Observational	India	PLT	26	6 Months	NR	NR
				HT	26			
Rhatomy et al. [8]	2019	Observational	Indonesia	PLT	24	12 Months	23.4	20
				HT	28		26.4	24
Saeed et al. [38]	2021	Observational	Pakistan	PLT	73	24 Months	NR	NR
				HT	85			
Sari et al. [39]	2025	Observational	Turkey	PLT	77	27 Months	27	77
				HT	82		26.6	82
Saugat et al. [40]	2025	Observational	Nepal	PLT	27	12 Months	NR	NR
				HT	27			
Shair et al. [41]	2022	RCT	Pakistan	PLT	35	9 Months	25.8	32
				HT	36		27.4	34
Shi et al. [42]	2019	RCT	China	PLT	18	24 Months	NR	NR
				HT	20			
Soni et al. [43]	2026	Observational	India	PLT	25	6 Months	37.2	19
				HT	25		36.2	21
Vijay et al. [44]	2022	RCT	India	PLT	23	12 Months	33.57	18
				HT	22		31.82	17
Vijay et al. [45]	2024	Observational	India	PLT	65	12 Months	30.38	NR
				HT	65		31.7	
Vyacheslavovich et al. [46]	2024	Observational	Russia	PLT	55	24 Months	32.9	38
				HT	55		33.1	41
Waly et al. [47]	2022	RCT	Egypt	PLT	25	12 Months	33.3	19
				HT	25		31.5	17

**Table 2:** Quality assessment of included studies References: [8,17-21,25,27-28,30-32,34-40,43,45-46]

Author	Selection	Comparison	Assessment	Overall
Acharya et al. [17]	4	2	2	Good
Agarwal et al. [18]	3	2	2	Good
Agarwal et al. [19]	2	1	2	Fair
Arva et al. [20]	2	1	2	Fair
Ashtekar et al. [21]	2	1	2	Fair
Choudhari et al. [25]	3	1	2	Fair
Gök et al. [27]	3	2	2	Good
Gunadham et al. [28]	4	2	2	Good
Kapoor et al. [30]	3	2	3	Good
Keyhani et al. [31]	4	2	3	Good
Khalid et al. [32]	3	1	3	Good
Ligu et al. [34]	4	2	2	Good
Nikumbh et al. [35]	2	2	2	Fair
Punnoose et al. [36]	3	2	2	Good
Rahul et al. [37]	4	2	2	Good
Rhatomy et al. [8]	3	2	2	Good
Saeed et al. [38]	4	1	2	Good
Sari et al. [39]	3	2	2	Good
Saugat et al. [40]	4	2	3	Good
Soni et al. [43]	2	2	2	Fair
Vijay et al. [45]	3	2	2	Good
Vyacheslavovich et al. [46]	4	2	2	Good

**Table 3:** Quality assessment of included randomized controlled trials (RCTs) References: [22-24,26,29,33,41-42,44,47]

Author	Randomization	Deviation	Missing Data	Outcome Measurement	Selective Reporting
Asif et al. [22]	Low Risk	Some Concerns	Low Risk	Some Concerns	Some Concerns
Bi et al. [23]	Low Risk	Low Risk	Low Risk	Low Risk	Some Concerns
Butt et al. [24]	Low Risk	Some Concerns	Some Concerns	Some Concerns	Low Risk
Dwidmuthe et al. [26]	Low Risk	Some Concerns	Low Risk	High Risk	Low Risk
Hussain et al. [29]	Low Risk	Some Concerns	Low Risk	High Risk	Some Concerns
Khalil et al. [33]	Low Risk	Some Concerns	Some Concerns	High Risk	Some Concerns
Shair et al. [41]	Low Risk	Some Concerns	Some Concerns	High Risk	Some Concerns
Shi et al. [42]	Low Risk	Low Risk	Low Risk	Low Risk	Low Risk
Vijay et al. [44]	Low Risk	Some Concerns	Some Concerns	Some Concerns	Low Risk
Waly et al. [47]	Low Risk	Low Risk	Some Concerns	Low Risk	Low Risk

Primary Outcomes

**Figure 2:**
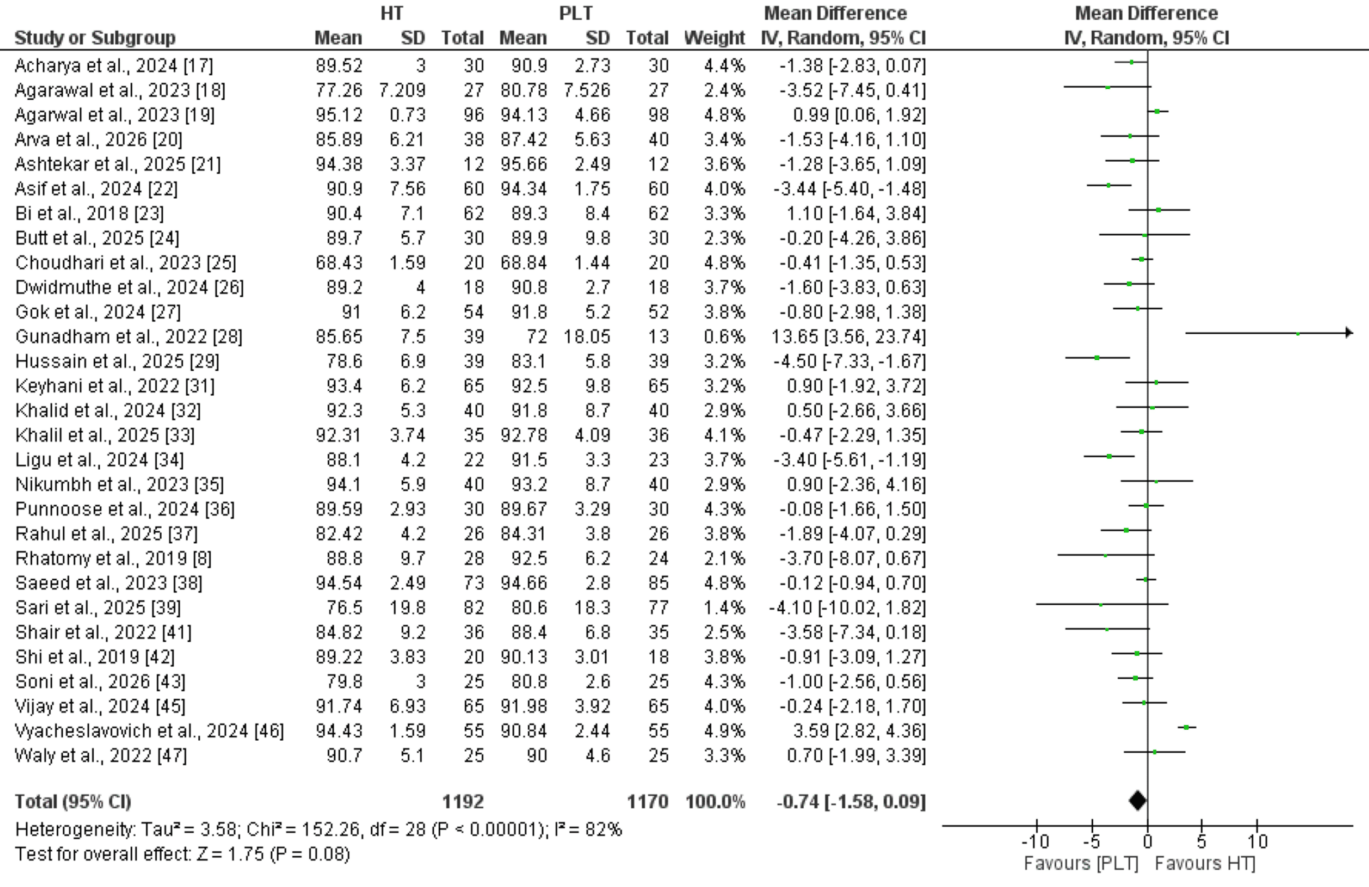
Comparison of International Knee Documentation Committee (IKDC) scores between the two groups References: [8,17-29,31-39,41-43,45-47]

IKDC score: A total of 29 studies were included in the pooled analysis comparing IKDC scores between hamstring and peroneus longus autografts (Figure 2). The pooled analysis demonstrated no statistically significant difference between the two graft types (MD: -0.74, 95% CI: -1.58 to 0.09), with high heterogeneity across studies (I² = 82%).

**Figure 3:**
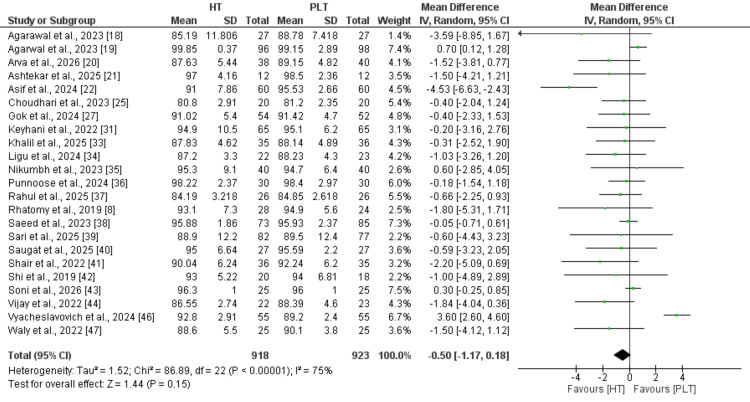
Comparison of Lysholm scores References: [8,18-22,25,27,31,33-44,46-47]

Lysholm score: A total of 23 studies were included in the pooled analysis comparing Lysholm scores between hamstring and peroneus longus autografts (Figure 3). No statistically significant difference was observed between the two graft types (MD: -0.50, 95% CI: -1.17 to 0.18), with high heterogeneity across studies (I² = 75%).

Subgroup Analysis of Primary Outcomes

Subgroup analysis was performed to compare the effect of hamstring and peroneus longus autografts on IKDC score and Lysholm score at six months, 12 months, and 24 months, and the results are presented in Table 4. In all follow-up points, neither of the outcomes was significantly different between the two groups. In the pooled analysis, we combined RCT and observational studies. Table 5 shows subgroup analysis based on study design. The observational studies’ pooled analysis did not show significant differences between the two groups in terms of IKDC and Lysholm scores. On the other hand, pooled analysis of RCTs demonstrated that IKDC and Lysholm scores are significantly lower in patients randomized in the hamstring group compared to the peroneus longus group.

**Table 4:** Subgroup analysis CI: confidence interval; IKDC: International Knee Documentation Committee; MD: mean difference

Outcomes	Groups	MD (95% CI)	I²
IKDC	6 Months	-1.64 (-2.73 to -0.55)	64%
	12 Months	-0.26 (-1.22 to 0.71)	58%
	24 Months	0.28 (-2.11 to 2.67)	45%
Lysholm Score	6 Months	0.10 (-0.74 to 0.93)	70%
	12 Months	-0.19 (-0.99 to 0.60)	68%
	24 Months	0.24 (-2.04 to 2.52)	52%

**Table 5:** Subgroup analysis (study design) CI, confidence interval; IKDC: International Knee Documentation Committee; MD, mean difference; RCT, randomized controlled trial

Outcome	Groups	MD (95% CI)	I²
IKDC	Observational	-0.10 (-1.14, 0.94)	66%
	RCT	-1.85 (-3.29 to -0.42)	54%
Lysholm Score	Observational	0.20 (-0.55 to 0.96)	62%
	RCT	-2.11 (-3.61 to -0.62)	49%

Secondary Outcomes

**Figure 4:**
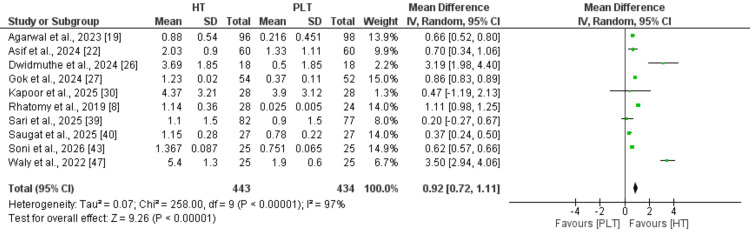
Comparison of thigh circumference between the two groups References: [8,19,22,26-27,30,39-40,43,47]

Thigh circumference: Eight studies were included in the pooled analysis to compare thigh circumference between hamstring and peroneus longus autografts. As shown in Figure 4, thigh circumference was significantly higher in patients in the hamstring group compared to the peroneus longus autografts (MD: 0.92, 95% CI: 0.72 to 1.11). High heterogeneity was reported among the study results (I² = 97%).

Other Outcomes

Table 6 presents the results of other outcomes. In the comparison between HT autograft and PLT autograft, no significant differences were observed in postoperative knee stability outcomes. For the anterior drawer test, the pooled OR was 1.14 (95% CI: 0.53-2.42; I² = 0%), indicating comparable anterior stability between the two graft types. Similarly, the Lachman test showed an OR of 2.01 (95% CI: 0.80-5.04; I² = 0%), suggesting no statistically significant difference. For the pivot shift test, the OR was 0.33 (95% CI: 0.01-8.21; I² = 0%), with a wide CI reflecting imprecision and no clear superiority of either graft. Additionally, the AOFAS score was not significantly different between the two groups (MD: 0.17, 95% CI: -0.06, 0.39). The I² value of 0% across all outcomes indicates no observed heterogeneity among the included studies. Overall, both HT and PLT autografts demonstrated comparable clinical stability outcomes.

**Table 6:** Comparison of secondary outcomes AOFAS, American Orthopaedic Foot and Ankle Society score; CI, confidence interval; OR, odds ratio *Presented as mean difference (MD) (95% CI)

Outcome	OR (95% CI)	I²
Anterior Drawer Test	1.12 (0.54 to 2.33)	0%
Lacman Test	2.01 (0.80 to 5.04)	0%
Pivot Shift Test	0.33 (0.01 to 8.21)	0%
Graft Failure	1.24 (0.55 to 2.80)	0%
Graft Diameter	-0.69 (-0.86 to -0.52)	91%
AOFAS Score*	0.17 (-0.06 to 0.39)	18%

Discussion

This meta-analysis of comparative studies was conducted to assess and contrast clinical outcomes following ACLR using PLT autografts versus HT autografts. Overall, the pooled results demonstrated no significant differences between the two graft types with respect to IKDC and Lysholm scores. In terms of thigh muscle atrophy, however, the PLT group exhibited significantly less reduction in thigh circumference compared with the HT group. Although the PLT group showed slightly lower AOFAS scores than the HT group, this difference did not reach statistical significance. Subgroup analysis according to follow-up duration revealed that IKDC scores were significantly higher in the PLT group at six months. These findings align with the results reported by Opoku et al. [48]. Notably, our analysis incorporated recently published RCTs and observational studies, strengthening the currency of the evidence.

The IKDC score is widely used to evaluate functional recovery after ACLR. In our analysis, PLT grafts were associated with a mean increase of 0.59 points compared with HT grafts overall, and a 1.64-point advantage at six months in subgroup analysis. Although the six-month difference reached statistical significance, its magnitude remains well below the reported minimal clinically important difference (MCID) of 9-20 points [49], limiting its clinical relevance given the subjective nature of this patient-reported outcome.

With respect to objective knee stability, pooled analyses of the anterior drawer test (OR: 1.12, 95% CI: 0.54-2.33), Lachman test (OR: 2.01, 95% CI: 0.80-5.04), and pivot shift test (OR: 0.33, 95% CI: 0.01-8.21) demonstrated comparable results between PLT and HT grafts. Heterogeneity was minimal (I² = 0%) across all stability measures, enhancing confidence in these findings. Restoration of knee stability remains the principal objective of ACLR and a key determinant of functional success [50]. The Lachman test, in particular, is considered the most sensitive clinical examination for ACL insufficiency, with reported sensitivity of 85-100% and specificity of 94-99% [51]. A negative postoperative Lachman test is widely regarded as a reliable indicator of restored anterior tibial stability and successful graft integration [52]. Collectively, these findings suggest that PLT autografts provide biomechanical stability equivalent to the well-established HT graft.

The growing clinical adoption of the PLT is strongly supported by its favorable biomechanical profile. The native ACL has a reported strength of 1725 ± 269 N, while the PLT demonstrates comparable properties, with an ultimate tensile strength of approximately 1950 N [9]. Even more notably, Rudy et al. [53] reported a PLT tensile strength of 2500 N, suggesting its load-bearing capacity may exceed that of the native ligament. These figures are clinically significant, as they indicate that the PLT not only meets but potentially surpasses the mechanical demands placed on a reconstructed ACL during functional activities. Cadaveric comparative studies found no statistically significant difference in tensile strength between the PLT and HT (p > 0.05) [23], and this equivalence is reinforced by in vivo data: in a cross-sectional biomechanical study of 51 patients, the PLT demonstrated significantly greater tensile strength than the HT, while graft diameters between the two were not significantly different.

An important but often underappreciated consideration in graft selection is the preservation of knee proprioception. ACL remnants have been shown to contain proprioceptive fibers that could enhance functional recovery if they adhere to or grow into the reconstructed ligament, and remnant-sparing surgical techniques have demonstrated improved outcomes and functional recovery compared to conventional complete debridement [54]. A critical advantage of PLT harvest is that it occurs entirely distal to the knee joint, leaving periarticular mechanoreceptors undisturbed. HT harvest frequently disrupts the hamstring-quadriceps balance, potentially compromising dynamic knee stability during rehabilitation; by contrast, PLT harvest minimizes local knee morbidity, with the peroneus brevis compensating for its loss in ankle eversion and plantarflexion [55]. When using PLT grafts, several surgical teams have adopted remnant-preservation strategies to further maximize proprioceptive recovery. Patients in whom a longer ACL stump (≥ 1/2 of original length) was retained during PLT-based reconstruction demonstrated better proprioception recovery and a higher proportion of return to exercise at short-term follow-up, with more complete synovial coverage reducing mechanoreceptor loss and promoting angiogenesis and ligamentization of the tendon graft [56].

One of the most clinically meaningful findings of this meta-analysis was the significant preservation of thigh circumference in the PLT group, with a MD of 0.92 cm compared with the HT group. Unlike small differences observed in patient-reported scores, this objective parameter carries genuine functional importance. Harvesting of HTs has long been associated with persistent flexor muscle weakness and atrophy [51]. Previous studies have shown that hamstring strength deficits ranging from 8% to 26% may persist for years despite structured rehabilitation [52]. Xergia et al. [57] reported significantly lower eccentric hamstring strength at four, 12, and 24 months postoperatively in patients receiving HT grafts compared with patellar tendon grafts. Similarly, Nakamura et al. [58] observed persistent knee flexor weakness of approximately 10% at a mean follow-up of 28 months after hamstring harvest. Such deficits may have important implications for athletes engaged in sports requiring rapid acceleration, deceleration, and directional changes [59].

In recent years, PLT autografts have gained recognition as a viable and non-inferior alternative to HT grafts. This growing interest is largely attributed to the lower incidence of knee donor site complications associated with PLT harvest [60]. Donor site morbidity following hamstring harvest, including thigh hypotrophy and quadriceps-hamstring imbalance, has emerged as a significant concern, contributing to increased consideration of PLT grafts [61].

Our analysis also demonstrated no significant difference in graft failure rates between PLT and HT groups, suggesting that graft diameter alone does not determine surgical success. Future investigations should further explore the complex relationship between graft size, mechanical stability, and biological incorporation to refine graft selection strategies.

Although reduced knee donor site morbidity was observed in the PLT group, concerns have been raised regarding potential ankle dysfunction after PLT harvest. The peroneus longus plays an essential role in ankle eversion and plantar flexion, contributing to lateral ankle stability and dynamic foot mechanics [62-63]. Biomechanical studies indicate that the peroneus longus accounts for approximately 60-65% of ankle eversion torque, with the peroneus brevis contributing the remaining 35-40% [64-65]. Consequently, theoretical concerns have centered on possible eversion strength deficits and compromised lateral ankle stability, particularly during high-demand activities [66]. Early reports suggested that complete PLT harvest might result in measurable eversion weakness ranging from 8% to 22%, depending on harvest extent and compensatory adaptation [67-68].

However, the pooled AOFAS results in our meta-analysis provide reassuring clinical evidence. No significant difference was observed between PLT and HT groups (MD: 0.17, 95% CI: -0.06 to 0.39, p > 0.05), and the magnitude of difference was far below the established MCID of 9 points [69]. This indicates that ankle function, as measured by validated outcome tools, remains clinically comparable following PLT harvest. Additionally, studies reporting low complication rates and high satisfaction further support the safety of PLT graft use. For example, Asif et al. [22] reported no ankle-related complications at two-year follow-up in 62 patients, with AOFAS scores exceeding 99 points in both PLT and comparison groups.

The strengths of this meta-analysis include (1) inclusion of a large number of studies (30 studies) with substantial cumulative sample size; (2) restriction to comparative designs to minimize bias; and (3) comprehensive evaluation of multiple clinical, functional, and stability-related outcomes.

Limitations

Several limitations of the present meta-analysis must be acknowledged when interpreting its findings. First, considerable variability existed among included studies regarding design, demographic characteristics (age, sex, injury-to-surgery interval), surgical techniques (harvest and fixation methods), and rehabilitation protocols, potentially introducing clinical heterogeneity and residual confounding. Second, statistical heterogeneity was significant for several outcomes, which may have reduced the precision and power of pooled estimates. Third, the follow-up periods across included studies were variable, and the long-term durability of PLT grafts beyond two to five years remains insufficiently characterized. Graft remodeling, ligamentization, and the development of late osteoarthritis are biological processes that unfold over extended periods, and short follow-up windows may not capture these outcomes adequately. Fourth, the majority of included studies originated from Asian populations, which may limit the generalizability of findings to other ethnic and demographic groups, given known variations in tendon morphology and body anthropometry. Fifth, outcome assessors were not consistently blinded across studies, introducing potential for performance and detection bias. Finally, none of the included studies formally assessed proprioceptive recovery or neuromuscular function as a primary outcome, representing a notable gap given the emerging evidence linking periarticular innervation to functional knee stability.

Future directions

The findings of this meta-analysis establish a foundation for several important avenues of future investigation. Well-designed, multicenter RCTs with standardized surgical protocols, rehabilitation regimens, and long-term follow-up (minimum five to 10 years) are needed to confirm the durability of PLT autografts relative to HT grafts. Future studies should incorporate objective neuromuscular and proprioceptive assessments, including threshold to detection of passive motion and joint position sense testing, to characterize the sensory recovery profile specific to PLT reconstruction. Research examining the interaction between PLT grafts and remnant-preservation techniques - and their combined effect on proprioceptive outcomes - would be particularly valuable. Additionally, investigations into graft ligamentization kinetics using MRI-based signal intensity analysis could help clarify the biological incorporation timeline of PLT grafts and inform return-to-sport protocols. Subgroup analyses stratifying outcomes by graft diameter, fixation technique, patient activity level, and sex are also warranted, as these variables may moderate the functional trajectory of reconstruction. Finally, health economic analyses comparing the total costs - including rehabilitation burden and donor-site morbidity - of PLT versus HT reconstruction would assist in informing evidence-based graft selection guidelines across diverse healthcare systems.

## Conclusions

In conclusion, this systematic review and meta-analysis demonstrate that PLT autograft represents a viable and effective alternative to HT autograft for primary ACLR. Both grafts demonstrated comparable functional outcomes, as reflected by similar IKDC and Lysholm scores, alongside equivalent objective knee stability across anterior drawer, Lachman, and pivot shift assessments. Notably, PLT autograft was associated with significantly greater preservation of thigh circumference, indicating reduced donor site muscle atrophy compared to HT harvest. Ankle function remained clinically unaffected following PLT harvest. These findings support PLT autograft as a safe and reliable graft choice, particularly when minimizing knee-related donor site morbidity is a clinical priority. Future well-designed RCTs with longer follow-up periods are warranted to further validate these conclusions.
